# Severe post-traumatic Pancreatoduodenal injury with septic shock and enteric fistula: A multidisciplinary challenge – Case report

**DOI:** 10.1016/j.tcr.2026.101384

**Published:** 2026-05-21

**Authors:** Lamiae Bennis, Adil Habbab, Soumaya Alj, Ridouan Benomar Benelkhaiat, Mohammed Khallouki

**Affiliations:** Anesthesia and Intensive Care Department, Faculty of Medicine and Pharmacy, Cadi Ayyad University, Marrakech, Morocco

**Keywords:** Pancreatoduodenal trauma, Damage control surgery, Septic shock, Enteric fistula, Multidrug-resistant infection, Trauma critical care

## Abstract

Severe pancreatoduodenal trauma presents complex management challenges with high morbidity and mortality. We report a case of 16-year-old male sustained severe pancreatoduodenal injuries following a high-velocity motorcycle collision, presenting with pancreatic contusion and partial duodenal transection. The postoperative course was complicated by septic shock, acute kidney injury, and subsequent development of a high-output duodenal fistula with multidrug-resistant infections. Through coordinated critical care, targeted antimicrobial therapy, and nutritional support, the patient demonstrated clinical improvement with resolution of major complications, ultimately achieving sufficient stability for ICU discharge. However, a significant functional impairment persisted requiring ongoing care. This case highlights the critical importance of prompt recognition of combined pancreatoduodenal injuries, staged surgical intervention, and multidisciplinary management in high-energy trauma.

## Introduction

Severe post-traumatic pancreatoduodenal injuries represent a significant management challenge in trauma surgery and critical care, demanding a multidisciplinary approach to mitigate high mortality. These complex injuries, which often involve a destructive combination of pancreatic parenchymal disruption and duodenal perforation, are typically caused by high-energy deceleration mechanisms such as motorcycle collisions and auto-pedestrian accidents. The resultant direct compression of the upper abdomen against the spinal column inflicts devastating damage to this critical region. The subsequent clinical course is perilous, characterized by a high risk of life-threatening complications including hemorrhagic shock, anastomotic failure, intra-abdominal sepsis, and the development of enteric fistulas.

Despite advances in trauma systems, these complications collectively contribute to persistently high mortality rates, often exceeding 30% even in contemporary series [1]. This elevated morbidity stems not only from the initial injury pattern but also from the profound systemic inflammatory response and catabolic state it instigates. Consequently, the management paradigm has evolved to recognize that surgical intervention, while crucial, is merely the initial phase of care. Ultimate success is contingent upon seamless integration with advanced critical care support, including targeted antimicrobial therapy, meticulous fluid and electrolyte management, and proactive nutritional strategies.

This report details the management of a 16-year-old male who sustained severe combined pancreatoduodenal injuries following a high-velocity motorcycle collision. His subsequent course serves as an illustrative case of the high-stakes decision-making, the necessity for staged surgical principles, and the indispensable role of sustained multidisciplinary coordination especially with the absence of consensus guidelines.

## Case presentation

A 16-year-old male with no significant medical history presented to the emergency department one hour after a severe trauma. The mechanism was a high-velocity motorcycle collision following by his ejection into a tree. On arrival, the patient was fully conscious (GCS 15), respiratory and hemodynamically stable (Respiratory Rate at 18, SpO2 = 98% room air, Blood Pressure at 134/80 mmHg) but tachycardic at 140 bpm with diffuse abdominal tenderness on examination.

**Fig. 1:**
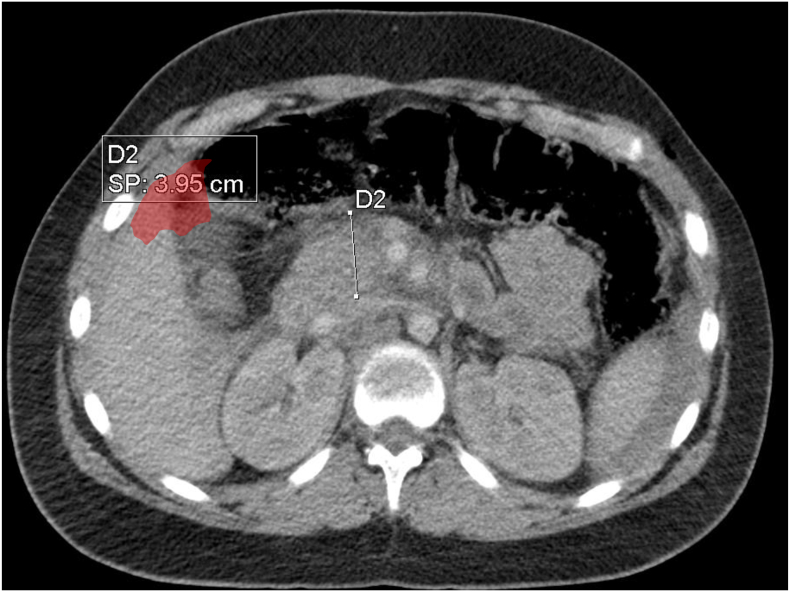
Axial computed tomographic section demonstrating swollen pancreas in its cephalic portion, measuring 39 mm in maximal diameter, corresponding on contusion grade III.

**Fig. 2:**
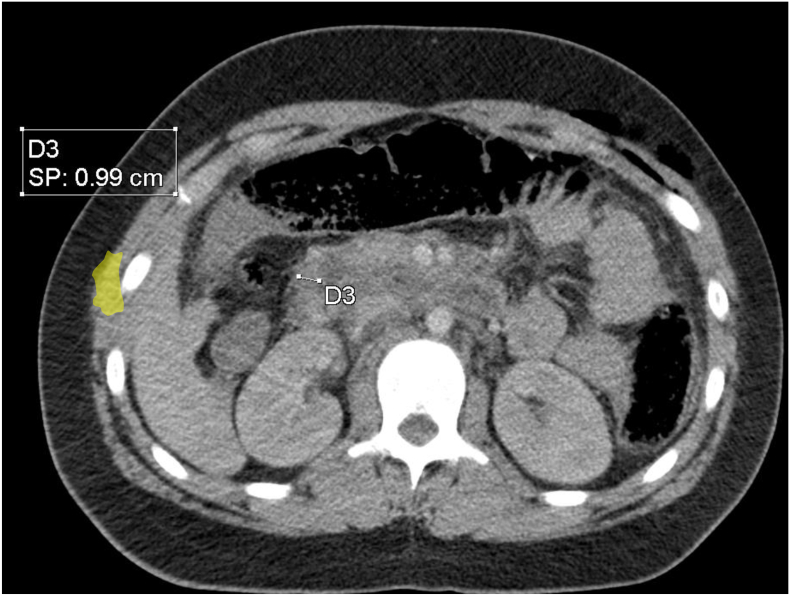
Axial computed tomographic section demonstrating circumferential wall thickening of the duodenum on his third portion (D3).

Initial CT imaging revealed a small pneumoperitoneum and moderate hemoperitoneum. Swollen pancreas in its cephalic portion, measuring 39 mm in maximal diameter, exhibiting a hypodense area on non-contrast imaging consistent was found, which was consistent with a grade III contusion (Fig. 1), with circumferential wall thickening of the duodenum (Fig. 2), raising suspicion for a hollow viscus injury.

The patient's condition rapidly progressed to hemodynamic instability, prompting immediate resuscitation with a norepinephrine infusion and initiation of broad-spectrum antibiotics (piperacillin-tazobactam and amikacin). Due to his worsening status, he was taken urgently for exploratory laparotomy.

**Fig. 3:**
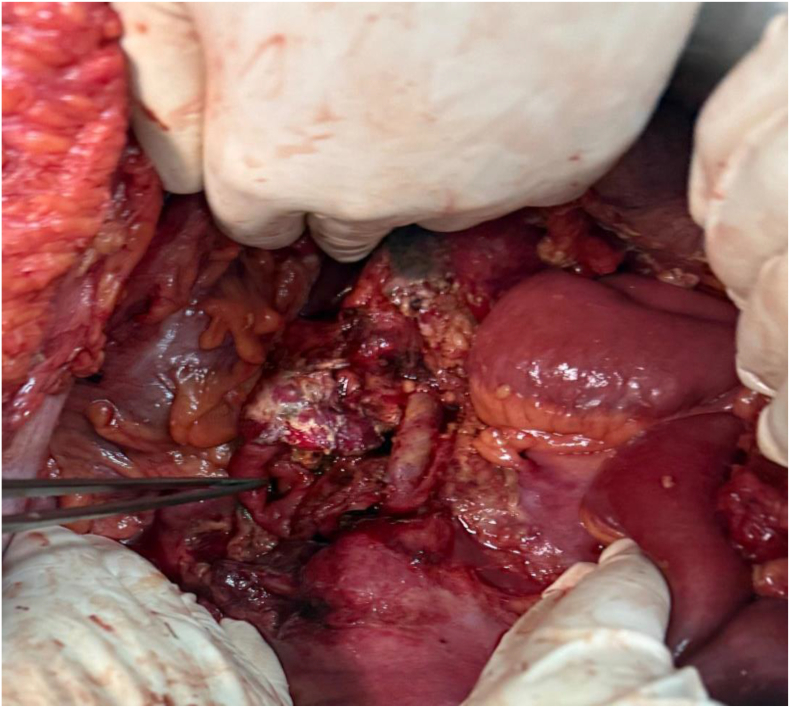
A peroperative transaction of the third part of duodenum, a pancreatic contusion, and fat stranding consistent with acute post-traumatic pancreatitis.

Under general anesthesia with rapid-sequence induction, surgical exploration revealed a significant hemoperitoneum, along with a cephalic pancreatic contusion that obscured definitive identification of a Wirsung duct injury. Multiple areas of fat necrosis were observed, consistent with post-traumatic pancreatitis (Fig. 3). Additionally, there was a bilateral anterolateral subcutaneous hematoma above the fascial layer and a partial transection of the third portion of the duodenum adjacent to the pancreatic contusion. The colonic framework, liver, and spleen exhibited no abnormalities.

Given the patient's instability, the surgical intervention included colic-epiploic detachment and mobilization of the right colic angle, followed by a Kocher maneuver. The duodenal injury was managed via primary repair using interrupted 3/0 Vicryl sutures (round needle) over a nasojejunal tube, with adequate hemostasis achieved. The abdomen was irrigated and drained with four suction drains: right and left subphrenic, bilateral Douglas pouch, and two Delbet drains near the duodenal suture line. Closure was performed in a layered fashion. The patient required substantial blood product resuscitation, receiving 4 units of packed red blood cells and 2 units of fresh frozen plasma intraoperatively.

The postoperative course was complicated by septic shock. The blood assessment revealed a CRP at 435 mg/L, Lactate concentration at 1.84 mmol/L, serum lipase levels at 189 units/L, creatine kinase levels at 29181 IU/L and acute kidney injury stage 3 of KDIGO. The management was based on the pursuit of resuscitation, renal protective measures, and antibiotics.

The evolution was marked by a gradual improvement in clinical status with hemodynamic stability, weaning of vasoactive drugs and return of diuresis. A concurrent improvement in biological parameters was noted (Table 1). The patient was extubated successfully at post operative day 8, and enteral feeding was initiated through the nasoenteric tube to support nutritional requirements.

**Table 1:** The evolution of biological assessment.

PO DAY	WBC (x10^3/μL)	CRP (mg/L)	Procalcit(ng/mL)	Hgb (g/dL)	Plt (x10^3/μL)	CK (IU/L)	Urea (g/L)	Creatinine (mg/L)	Serum Lipase (IU/L)
D2	12.4	261	–	12.8	230		0.89	25.7	189
D3	14.9	453	–	11	179	29,181	1.39	48.6	133
D4	15.4	375	–	9.6	147	19,636	1.65	63.7	113
D5	13.6	277	–	8.3	130	8590	1.4	62	–
D7	12.0	164	–	8.2	207	–	1.6	48	90
D9	15.1	127	–	7.7	338	–	1.32	62.0	–
D11	19.9	106	–	8.5	413	–	0.9	30.1	–
D13	21.0	159.9	3.34	7.2	218	–	0.74	20.0	–
D15	21.0	159	3.01	8.2	337	–	0.63	16.0	87
D17	16.6	110.6	0.76	8.2	240	38	0.66	15.0	–
D19	14.0	98.1	0.62	7.2	183	–	0.57	11.0	–

WBC: White Blood Cells / CRP: C-Reactive Protein/ Procalcit: Procalcitonin.

Hgb: Hemoglobin/ Plt: Platelet count/ CK: Creatine kinase.

**Fig. 4:**
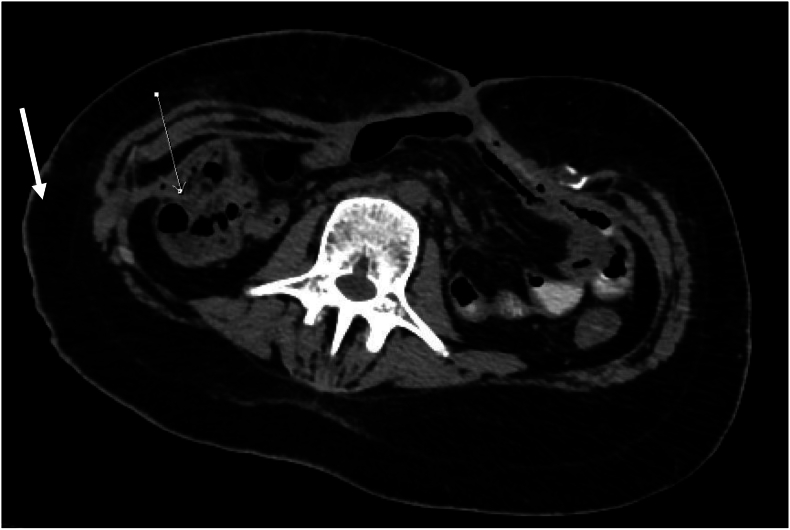
Axial computed tomographic section demonstrating an opacification of the left colon without opacification of the ascending colon, with passage of the contrast medium through the duodenocolic fistula.

However, a complication emerged on postoperative day 13 in the form of intractable vomiting, intermittent fever and a high-output (500 mL/day) secondary to a duodenoperitoneal fistula revealed by the opacification of the left colon without opacification of the ascending colon, due to passage of the contrast medium through the duodenocolic fistula (Fig. 4). Procalcitonin was at 3.3 ng/L and the microbiological culture results confirmed a positive pus culture for methicillin-resistant coagulase-negative Staphylococcus, a multidrug-resistant *Escherichia coli* and *Klebsiella pneumoniae* susceptible only to tigecycline and high-dose imipenem.

Given the controlled, well-drained nature of the fistula, the absence of diffuse peritonitis, and the high risk of reoperation in the setting of ongoing sepsis and friable tissues, a conservative management strategy was pursued. This included targeted antibiotics (vancomycin, imipenem, and tigecycline) and continued drainage. Total parenteral nutrition was initiated on post operative day 9 due to high-output fistula and intractable vomiting, transitioning to progressively increased jejunal feeds via nasoenteric tube.

By postoperative day 20, the patient demonstrated major clinical improvement which allowed him to be transferred to the visceral surgery department for ongoing care.

## Discussion

Our discussion will focus on the various challenges encountered in the management of severe Our discussion will focus on the various challenges encountered in the management of severe pancreatoduodenal trauma. The clinical course of our 16-year-old patient demonstrates both typical and unique features of this complex injury, offering valuable insights for trauma teams.

### Diagnostic challenges

The diagnostic complexities highlighted in our patient's case-initially subtle CT findings (duodenopancreatic contusion) followed by rapid clinical deterioration-mirror the broader challenges outlined by Stawicki et al. [2]. Their work emphasizes that pancreatic injuries are frequently missed on initial imaging, particularly in blunt trauma, due to the organ's retroperitoneal location and the predominance of concomitant injuries (e.g., duodenal, vascular) that divert clinical attention [2], [3]. In our patient, the small pneumoperitoneum and hemoperitoneum were the only early radiographic clues, underscoring the authors' observation that CT sensitivity is highly variable (47–80%) and often fails to detect ductal injuries without parenchymal disruption [2], [4].

The progressive nature of pancreatic injury further complicates diagnosis. Stawicki & Schwab describe a “dormant period” followed by systemic collapse, akin to our patient's delayed hemodynamic instability and subsequent septic shock. This aligns with their finding that serum amylase/lipase lack reliability (normal in 29% of blunt trauma cases even with ductal transection), reinforcing the need for serial clinical reassessment over biochemical markers [2], [5].

The cephalic pancreatic contusion with obscured ductal integrity encountered during laparotomy reflects the authors' emphasis on direct visualization as the gold standard for diagnosis [2], [6]. Their data show that 50% of pancreatic injuries require surgical exploration for definitive grading, particularly when CT reveals >50% parenchymal laceration or peripancreatic fluid [2], [7]. Our choice of primary duodenal repair with wide drainage aligns with their recommended approach for Grade II-III injuries, where ductal involvement is uncertain [2], [8]. Notably, the fat necrosis and evolving pancreatitis observed intraoperatively correlate with their warning that enzymatic autodigestion can exacerbate local and systemic injury [2], [9].

### Surgical management

The surgical management of pancreatic trauma is guided by injury severity, hemodynamic stability, and ductal integrity, as outlined by the American Association for the Surgery of Trauma (AAST) classification system. For low-grade injuries (AAST I-II), conservative management with drainage may suffice, whereas high-grade injuries (AAST III-V) often require operative intervention [7], [10]. Distal pancreatectomy (with or without splenectomy) remains the standard for ductal injuries in the body or tail, while complex proximal injuries may necessitate techniques like Roux-en-Y pancreaticojejunostomy or, in rare cases, pancreaticoduodenectomy (Whipple procedure) [1], [10]. Notably, the Whipple procedure is reserved for devastating injuries involving the pancreatic head, duodenum, or ampulla, as its morbidity and mortality (30–40%) are prohibitive in unstable trauma patients [10], [11].

A critical consideration in surgical planning is the principle of damage control surgery (DCS), which prioritizes hemorrhage control and contamination reduction over definitive repair in hemodynamically unstable patients [12]. Staged laparotomies allow for physiological stabilization before complex reconstructions, reducing mortality associated with prolonged initial surgeries [12], [13]. Additionally, the role of endoscopic retrograde cholangiopancreatography (ERCP) has expanded beyond diagnosis to therapeutic stenting for ductal disruptions, potentially avoiding surgery in select cases [14].

### Fistula management

The management of enteral fistulas, particularly duodenal fistulas (DF), in the context of pancreatic trauma remains a significant clinical challenge, with treatment strategies ranging from conservative measures to aggressive surgical interventions. In our case, a conservative approach was employed, focusing on antimicrobial therapy and nutritional support, which aligns with evidence suggesting that selected patients with stable conditions and controlled infections may avoid immediate surgical intervention. Leppäniemi et al. [15] reported that while surgical closure with duodenal decompression offers the highest success rates (76% fistula closure in their cohort), nonoperative management (NOM) can still be effective in carefully selected cases, achieving closure in 83% of patients treated conservatively. However, the same study highlights that failed NOM often necessitates delayed surgery, underscoring the importance of close monitoring for signs of clinical deterioration, such as persistent sepsis or peritonitis.

The conservative approach in our patient prioritized infection control and metabolic support, mirroring principles from other studies where nutritional optimization and percutaneous drainage played key roles in fistula resolution [16]. Notably, Leppäniemi et al. observed that patients with DF secondary to pancreatic pathology (severe acute pancreatitis) had higher mortality rates (40% overall), emphasizing the need for early recognition of complications like multiorgan failure or gastrointestinal bleeding [15]. While our patient avoided surgical intervention, the literature suggests that surgical repair-particularly resection with anastomosis or decompressive techniques-may be required if conservative measures fail, especially in high-output fistulas or those associated with systemic sepsis [17].

Future studies should explore biomarkers (IL-6, procalcitonin) to stratify patients likely to benefit from NOM versus early surgery [18]. Our case reinforces that conservative management is viable in stable patients, but clinicians must remain vigilant for indications of failure, such as persistent infection or clinical decline, which warrant prompt surgical evaluation.

### Antimicrobial considerations and nutrition

The isolation of multidrug-resistant organisms (MR-CoNS, ESBL-*E. coli*, and *K. pneumoniae*) reflects emerging antimicrobial challenges in trauma care. Our antibiotic selection adhered to current Infectious Diseases Society of America (IDSA) guidelines for abdominal trauma-associated infections [19], highlighting the importance of antimicrobial stewardship in this vulnerable population.

Nutrition in the postoperative period constitutes a real challenge for the medical staff since enteral feeding must be stopped to allow anastomosis healing, while at the same time patients need a hypercaloric diet to allow better consolidation and healing of anastomoses. The ultimate solution is parenteral nutrition, allowing to overcome this period while waiting for surgeons to authorize the resumption of enteral feeding [20].

The management of duodenopancreatic trauma often necessitates nutritional support, with parenteral nutrition (PN) playing a critical role in patients unable to tolerate enteral feeding due to fistulas, postoperative ileus, or high-output leaks [2]. In our case, PN was initiated postoperatively to meet caloric and protein demands while allowing pancreatic and duodenal healing. However, prolonged PN carries risks, including catheter-related bloodstream infections, metabolic complications, and intestinal mucosal atrophy [2], [21]. Transitioning to enteral nutrition (EN) is prioritized once gastrointestinal function permits, as EN preserves gut barrier integrity, reduces infectious complications, and supports immune function [2], [22]. Our patient's transition to EN by postoperative day 20 aligns with evidence suggesting that early EN-when feasible-improves outcomes in complex pancreaticoduodenal trauma [2], [23].

The article by Stawicki and Schwab (2008) emphasizes that PN should be reserved for patients with contraindications to EN (persistent fistulas, duodenal discontinuity) or those requiring damage control surgery [2], [21]. In such cases, PN is typically initiated by postoperative days 5–7 if EN remains unviable [2]. For our patient, the delayed transition to EN likely reflected the severity of injury and need for fistula stabilization. Notably, the authors highlight that EN via jejunostomy or nasojejunal tubes is preferable for distal feeding, as it bypasses the duodenum and minimizes pancreatic stimulation [2], [22]. Future studies should explore biomarkers (prealbumin, CRP) to optimize PN-to-EN transition timing in high-risk patients [24].

## Limitations and generalizability

It is important to acknowledge that the generalizability of our experience is constrained by several factors. Firstly, as a case report, its single nature means the successful outcome with conservative management may not be universally applicable, especially for older patients with significant comorbidities. Secondly, the outcome was heavily influenced by patient-specific factors; the resilience of a healthy 16-year-old likely contributed to success, a scenario that would be less probable in an immunocompromised or elderly patient who might require earlier intervention. Lastly, this approach hinges on institutional expertise, including advanced interventional radiology, a specialized multidisciplinary ICU team, and surgical readiness-resources whose variable availability across centers affects outcomes.

## Conclusions

This case of high-grade pancreatoduodenal trauma underscores the success of a systematic, multidisciplinary approach. Adherence to damage control principles including staged surgery, aggressive resuscitation, and meticulous critical care was paramount in managing severe complications like septic shock and a high-output enteric fistula.

While this case provides a successful management template, the inherent complexity and rarity of such injuries highlight that definitive guidelines cannot be drawn from single-institution experiences. Therefore, future priorities must include developing more sensitive imaging protocols for ductal injury, standardizing antimicrobial stewardship, and generating evidence-based fistula management algorithms. To validate these strategies and develop personalized care approaches, future research must focus on aggregating multi-center data for this high-risk population.

## CRediT authorship contribution statement

**Lamiae Bennis:** Writing – review & editing, Writing – original draft, Visualization, Validation. **Adil Habbab:** Writing – review & editing, Writing – original draft, Visualization, Validation. **Soumaya Alj:** Writing – review & editing, Writing – original draft, Visualization, Validation. **Ridouan Benomar Benelkhaiat:** Writing – review & editing, Writing – original draft, Visualization, Validation. **Mohammed Khallouki:** Writing – review & editing, Writing – original draft, Visualization, Validation.

## Declaration of competing interest

The authors declare that they have no known competing financial interests or personal relationships that could have appeared to influence the work reported in this paper. All authors certify that they have no affiliations with or involvement in any organization or entity with any financial interest (such as honoraria, educational grants, participation in speakers' bureaus, membership, employment, consultancies, stock ownership, or other equity interest, and expert testimony or patent-licensing arrangements) or non-financial interest (such as personal or professional relationships, affiliations, knowledge, or beliefs) in the subject matter or materials discussed in this manuscript.
